# 

**Published:** 1896-03-07

**Authors:** 


CONTENTS.

**The Ethics of Operations** . . . . . . . . . 373

**Infantile Diarrhœa and Artificial Feeding** . . . 374

**On Modern Progress in Ophthalmic Medicine and Surgery.**—Latent Squint (*continued*). By R. B. CARTER, M.D. 375

**Modern Sociology.**—Penology . . . . . . . 376

**Annotations.**—One Cause of Hospital Abuse—The "New Child" as "Made in Germany"—Mental Defects and Bodily Abnormalities . . . . . . . . . . . . 377

**Notes and News** . . . . . . . . . . 378

**Medical Progress and Hospital Clinics**—

Leg versus Arm Vaccination . . . . . . 379

PROGRESS IN MEDICINE.—Diseases of the Nervous System . 379

Diseases of the Stomach . . . . . . . . 382

THERAPEUTIC NOTES . . . . . . . . 384

NEW APPLIANCES AND THINGS MEDICAL . . . . . 384

**The Institutional Workshop**—

HOSPITAL MEETINGS . . . . . . . . . 385

EXTRA SUPPLEMENT.—THE NURSING MIRROR.

**News from the Nursing World.**—Royal Visits—London Homœopathic Hospital—Royal Free Hospital Medical School—East London Hospital for Children—Guy's Hospital Ladies' Association—A Pension Fund Nurse—A German University for Women—Queen's Nurses, Inverness Branch—Miss Blennerhassett in South Africa—Fire at Liverpool Hospital—The Trained Nurses' Club—University Degrees for Women—Queen's Jubilee Nurses and the Pension Fund—Short Items cxcvii

**Lectures on Nursing. XII.**—Observation . . . . cxcix

**Homeward Bound** . . . . . . . . . . cc

**Superintendents of Training Schools Association** cci

**Talks about Food.** VII.—Babes and Sucklings . . . ccii

**On Certain Aspects of the Nursing Question as seen in England and Germany** . . . . . cciii

**A Book and its Story** . . . . . . . cciv

**Everybody's Opinion** . . . . . . . . ccv

**Notes and Queries** . . . . . . . . ccvi

**Where to Go** . . . . . . . . . . ccvi

